# Factors related to sepsis and outcome in multiple trauma patients

**DOI:** 10.1186/cc14398

**Published:** 2015-03-16

**Authors:** H Pavlou, E Pappa, M Eforakopoulou

**Affiliations:** 1 KAT-EKA General Hospital, Kifisia, Athens, Greece

## Introduction

The outcome of multiple trauma patients is related to a number of diagnostic and therapeutic interventions during hospitalization. ICU patients with severe trauma are susceptible to sepsis leading to poor outcome. Factors associated with the occurrence of sepsis and the outcome of these patients were investigated.

## Methods

We studied retrospectively all trauma patients admitted to the A' ICU of KAT General Hospital in Athens during the last 3 years and were treated for more than 5 days. Age, gender, the type of injury, the severity of injury (Injury Severity Score), the length of ICU stay, severe sepsis, coexisting diseases, the outcome and the cause of death were recorded. Logistic regression and chi-square tests were used for statistical analysis.

## Results

A total of 106 multiple trauma patients, 85 men and 21 women, met the inclusion criteria. Depending on their age, patients were divided into two groups: <60 years old and >60 years old. In both groups, gender, the type and severity of injuries and the length of ICU stay were not associated with outcome. The length of ICU stay was correlated with severe sepsis and coexisting diseases (*P *< 0.01) in both groups. Mortality was not different in the two groups. The presence of at least one coexisting disease was significantly associated with mortality (*P *< 0.007). Sepsis was significant cause of death in trauma patients >60 years (*P *< 0.05).

## Conclusion

In multiple trauma patients, the length of ICU stay and comorbidities influence the occurrence of severe sepsis, comorbidities increase mortality, and sepsis is the leading cause of death in trauma patients >60 years old.
